# Editorial: Visualization and assessment of cerebral vasculature, cerebrospinal fluids, and the brain parenchyma in dementia and aging

**DOI:** 10.3389/fnagi.2024.1443028

**Published:** 2024-06-18

**Authors:** Jianpan Huang, Yachao Zhang, Lin Chen, Dengrong Jiang, Zixuan Lin

**Affiliations:** ^1^Department of Diagnostic Radiology, The University of Hong Kong, Hong Kong, China; ^2^Medical Ultrasound Department, Suzhou Institute of Biomedical Engineering and Technology, Chinese Academy of Sciences, Suzhou, China; ^3^Department of Electronic Science, Fujian Provincial Key Laboratory of Plasma and Magnetic Resonance, School of Electronic Science and Engineering, National Model Microelectronics College, Xiamen University, Xiamen, China; ^4^The Russell H. Morgan Department of Radiology and Radiological Science, Johns Hopkins University School of Medicine, Baltimore, MD, United States; ^5^Key Laboratory for Biomedical Engineering of Ministry of Education, Department of Biomedical Engineering, College of Biomedical Engineering and Instrument Science, Zhejiang University, Hangzhou, China

**Keywords:** dementia, brain, glymphatic system, cerebrospinal fluid, magnetic resonance imaging, photoacoustic (optoacoustic) imaging

The glymphatic system (meningeal lymphatic system) hypothesis proposes that the perivascular spaces (PVS) in the brain serve as channels for cerebrospinal fluid (CSF) to mix with interstitial fluid (ISF), aiding in the removal of waste and proteins from the brain (Iliff et al., 2012; Jessen et al., 2015). Recent research has established a link between this system and the clearance of Aβ and tau, both of which are connected to dementia. The proper functioning of the glymphatic system relies heavily on the aquaporin 4 water channel (AQP4) (Mestre et al., 2018). Deficiencies or absences of AQP4 can disrupt water transport, leading to various brain conditions. Medical imaging techniques can visualize and assess the cerebral vasculature, CSF, and their interactions with brain tissue, potentially aiding in the early detection and diagnosis of dementia (Huang et al., 2020, 2022; Taoka and Naganawa, 2020; Klostranec et al., 2021). By identifying abnormalities or disturbances in the glymphatic system, these imaging techniques offer valuable insights into the development and progression of dementia.

Enlarged PVS (EPVS) has been linked to the development of neurodegeneration. Elevated levels of AQP4 in CSF have also been found in patients with neurodegenerative dementia (Jeong et al., 2022). Sacchi et al. retrospectively examined the relationship between two potential biomarkers of the glymphatic system, EPVS and AQP4, and investigated CSF biomarkers associated with neurodegeneration in a population of individuals suspected to have dementia. Results revealed a significant correlation between EPVS, specifically in the centrum semiovale (CSO), and AQP4 levels in the CSF among patients undergoing neurological evaluations for suspected dementia. Additionally, the researchers observed that both the count of EPVS in the CSO and the levels of AQP4 in the CSF were associated with the levels of CSF-tTau, an established biomarker for neurodegeneration. CSO-EPVS and CSF-AQP4 could potentially serve as clinical biomarkers for glymphatic dysfunction and the subsequent development of neurodegeneration.

Cerebrovascular reactivity (CVR) has shown great promise in assessing neurodegenerative diseases, such as Alzheimer's disease (Silvestrini et al., 2006). However, CVR is typically assessed using a CO_2_ stimulus, which requires specific apparatus for CO_2_ delivery, limiting its clinical availability. To address this problem, resting-state CVR (rs-CVR) exploits the spontaneous fluctuations in arterial CO_2_ levels as the stimulus to generate CVR maps without the need for gas challenges. The study by Liu et al. demonstrated that good rs-CVR map quality can be obtained at a voxel size as small as 2 mm isotropic, suggesting that rs-CVR can be applied at a similar resolution to state-of-art fMRI studies. This study may broaden the use of rs-CVR in both basic science and clinical applications. It also opens the door for retrospective analyses of previously collected high-resolution fMRI data to obtain CVR, providing additional information about the patient's vascular health beyond traditional fMRI metrics such as functional connectivity.

Neuroimaging studies have indicated that individuals with Alzheimer's disease typically exhibit gray matter atrophy and white matter abnormalities, potentially indicating alterations in the gray-white matter boundary (Phillips et al., 2016). Tian et al. investigated differences in gray-white matter boundary Z-score (gwBZ) and its tissue volume (gwBTV) among Alzheimer's disease patients, individuals with mild cognitive impairment (MCI), and cognitively normal (CN) elderly participants. Utilizing 3D T1-weighted MRI images, the study mapped gwBZ and gwBTV and analyzed their correlations with cognitive function and age. The results indicated that Alzheimer's disease patients exhibited significantly lower gwBZ and gwBTV compared to CN and MCI groups. Moreover, gwBZ and gwBTV showed positive correlations with cognitive scores on the Korean version of the Mini-Mental State Examination (K-MMSE) and negative correlations with age. Combining gwBZ or gwBTV with K-MMSE enhanced the accuracy of Alzheimer's disease classification, with an area under the curve (AUC) value of 0.972. These findings suggest that evaluating gwBZ and gwBTV can be a valuable tool for diagnosing and monitoring the progression of Alzheimer's disease.

Coronary artery disease (CAD) is a cardiovascular condition caused by plaque buildup in the arteries, leading to narrowed or blocked blood vessels. This can reduce blood flow to the heart and cause heart-related issues and further affect brain health (Kovacic et al., 2012). Li et al. conducted a study using diffusion kurtosis imaging (DKI) to examine brain microstructure changes in CAD patients with cognitive impairment. The study found that CAD patients showed radial shrinkage and increased complexity in the white matter of the brain. By analyzing the entire white matter using tract-based spatial statistics (TBSS) DKI, researchers objectively identified characteristics of white matter damage in CAD patients. This information could potentially aid in the diagnosis of CAD in individuals with cognitive impairment.

Disproportionately enlarged subarachnoid-space hydrocephalus (DESH) is a prominent characteristic of Hakim disease (idiopathic normal pressure hydrocephalus: iNPH) (Adams et al., 1965). Yamada et al. have developed an automated method for quantitatively assessing DESH using 3D T1-weighted or T2-weighted MRIs. This method involves the utilization of two artificial intelligence (AI) models: a 3D U-Net for semantic segmentation and a multimodal convolutional neural network for image classification. The study results suggest that AI-based diagnostic imaging support, combined with quantitative assessment of DESH, could enhance the accuracy of diagnosing Hakim disease (iNPH) by reducing missed and misdiagnosed cases. Additionally, this approach shows potential for application in future multicenter collaborative studies.

In addition to MRI, researchers have explored other imaging methods for potential use in imaging brain diseases. Mi et al. conducted a review that focused on the potential of photoacoustic imaging in managing Alzheimer's disease. This imaging technique combines the benefits of optical imaging and ultrasound and has shown promise in preclinical studies for detecting subtle molecular and structural brain changes before the onset of clinical symptoms in Alzheimer's disease. Early detection could lead to improved patient outcomes through timely interventions. However, implementing these techniques in real-world clinical settings, where various factors can affect their effectiveness, poses a significant challenge. Therefore, while photoacoustic imaging offers hope in Alzheimer's disease research, further refinement and validation are necessary to ensure its applicability and potential benefits for a wider range of patients affected by this challenging disease.

## Author contributions

JH: Writing – original draft. YZ: Writing – review & editing. LC: Writing – review & editing. DJ: Writing – review & editing. ZL: Writing – review & editing.
